# Interfacial Polarization Mechanism in Image Sticking of Polyimide-Based Flexible OLEDs

**DOI:** 10.3390/polym17172333

**Published:** 2025-08-28

**Authors:** Zhipeng Li, Haowen Li, Dawei Ma, Baojie Zhao, Yanbo Li

**Affiliations:** 1 Institute of Fundamental and Frontier Sciences, University of Electronic Science and Technology of China, Chengdu 611000, China; 2 Mianyang BOE Optoelectronics Technology Co., Ltd., Mianyang 621000, China

**Keywords:** polyimide, image sticking, interface polarization

## Abstract

Organic light-emitting diodes (OLEDs) have emerged as a critical battleground in display technology due to their self-emissive and foldable properties. However, the adoption of polyimide (PI) as a flexible substrate material introduces technical challenges, particularly image sticking. This study proposes an interfacial polarization mechanism to explain this phenomenon, confirmed through dielectric and ferroelectric spectroscopy. The results show that introducing an amorphous silicon (α-Si) interlayer significantly improves interface compatibility, increasing the polarization response frequency from 74 Hz to 116 Hz and reducing residual polarization strength from 2.81 nC/cm^2^ to 1.00 nC/cm^2^. Practical tests on OLED devices demonstrate that the optimized structure (PI/α-Si/SiO_2_) lowers the image sticking score from 3.46 to 1.67, validating the proposed mechanism. This research provides both theoretical insights and practical solutions for mitigating image sticking in flexible OLED displays.

## 1. Introduction

Organic light-emitting diodes (OLEDs) have emerged as the critical battleground in display technology competition since LG and Samsung successively withdrew from LCD production [1]. Due to the self-emissive and foldable properties, OLEDs have already yielded numerous products, exemplified by Samsung’s Galaxy Fold series and Huawei’s foldable laptops [2]. These highly anticipated products benefit from the adoption of flexible substrates for thin film transistors (TFTs) [3]. Among various flexible polymers, polyimide (PI) has emerged as the most widely adopted base material due to its superior thermal stability and high optical transmittance [4].

However, this widespread adoption has also introduced several technical challenges. Image sticking is one of them, referring to the visible persistence of a previous image after screen content changes [5]. The checkerboard test pattern represents the most established evaluation method for this phenomenon. Interestingly, the phenomenon is quite slight when glass is used as OLED substrates [6,7]. Great efforts have been made to illustrate the mechanism between image sticking and PI. The researchers from Samsung reported that the residual fluoride ions (F^−^) in PI would move towards the interface under the external electric field, resulting in charge accumulation and causing threshold voltage (Vth) drifting [8,9]. Therefore they introduced SiCOH to compensate for the surface charging induced by F^−^ [10]. However, some researchers have also suggested that it is related to some positive ions, such as H ions, and the polarity conversion of charges is the reason for image sticking [11]. Although the mechanisms are different, there is no doubt that this issue is related to the stability or the hysteresis of TFT [12,13].

In this article, the polarization mechanism proposed for explaining image sticking is related to PI. The current back layer of TFT is mostly stacked by PI/SiO_2_ or Si_3_N_4_ [14]. There is a significant electrical difference between polymer and inorganic Si film. According to the theory of dielectrics, interface polarization will occur under the electric field, and space charges are generated [15,16]. We confirmed the existence of interface polarization using dielectric and ferroelectric spectroscopy. The dielectric results show that pure PI only exhibits dipole polarization, and PI/SiO_2_ shows interface polarization at low frequencies. Adding the amorphous silicon (α-Si) layer between PI and SiO_2_ to increase interface compatibility, the corresponding frequency can be increased from 74 Hz to 116 Hz. On the other hand, the residual polarization strength is influenced by frequency and external field strength, and the typical value is 2.81 nC/cm^2^ under the conditions of 50 MV/m and 0.1 Hz. When α-Si is introduced, it can be decreased to 1.00 nC/cm^2^. Ultimately, faster polarization response speed and less charge will result in better residual image performance in the actual products. This article proposes the interface polarization mechanism of image sticking and especially points out the influence of slow processes. Increasing interface compatibility can optimize this question, and the results will contribute to further optimizing and improving image sticking.

## 2. Materials and Methods

**Figure 1:**
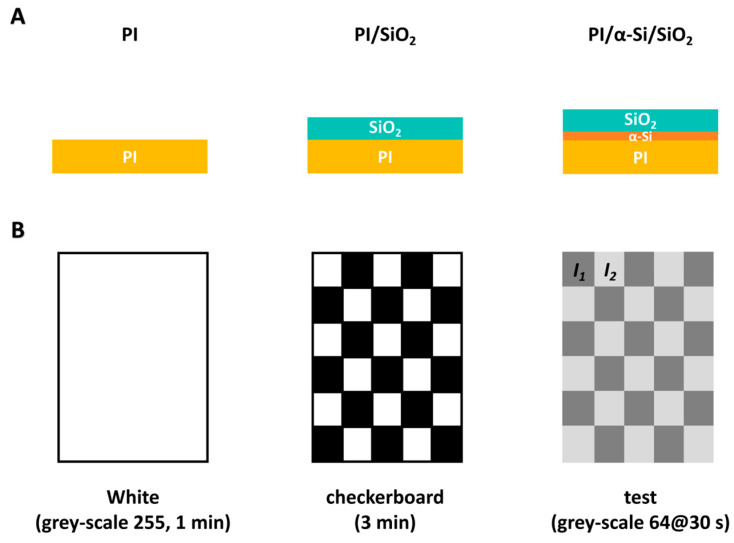
(**A**) The structure of three samples. (**B**) The tests procedure for image sticking.

The PI film is synthesized via gradient heating with polyamic acid (PAA) solution. The PAA solution is coated on the glass at first, then it is converted to polyimide (PI) via thermal imidization by vacuum thermal evaporation and programmed heating (200 °C for 1 h, 300 °C for 1 h, and 450 °C for 1 h). The structure of PI is shown in Figure S1. Both SiO_2_ and α-Si are deposited via plasma-enhanced chemical vapour deposition (PECVD), which is widely used in OLEDs due to lower temperatures and higher deposition rates. Three samples are marked as PI (5.8 μm), PI/SiO_2_ (5.8 μm/0.5 μm), and PI/α-Si/SiO_2_ (5.8 μm/0.005 μm/0.5 μm), shown as Figure 1A. Finally, the films are peeled off from the glass by laser. The structure is determined by Focus-Ion-Beam scanning electron microscopy (FIB-SEM, Crossbeam 350, ZEISS, Oberkochen, Germany). UV-vis and photoluminescence (PL) spectra are tested on a TU-1901 spectrophotometer (Beijing Purkinje General Instrument Co., Ltd., Beijing, China) and FLS920 fluorescence spectrometer (Edinburgh Instruments, Livingston, UK). For dielectric and ferroelectric tests, Ag electrodes, about 2 mm in diameter, are deposited by evaporation with specific models. Dielectric curves are tested on DMS 500 (BALAB, Wuhan, China). Ferroelectric data are recorded on TF Analyzer 3000 (aixACCT, Aachen, Germany).

For image sticking tests, the traditional black-and-white checkerboard method is employed at 25 °C (Figure 1B) [8]. First, a white image (grey-scale 255) is displayed for 1 min to establish stable baseline values. Next, the checkerboard pattern is displayed for 3 min to induce interface polarization and cause image sticking. Finally, the image changes to a grey-scale of 64, and the average brightness values, *I*_1_ and *I*_2_, are recorded at 30 s. The image sticking is evaluated using the formula |(*I*_1_ − *I*_2_)/(*I*_1_ + *I*_2_)|/0.004. A higher result indicates more pronounced image sticking.

## 3. Results

**Figure 2:**
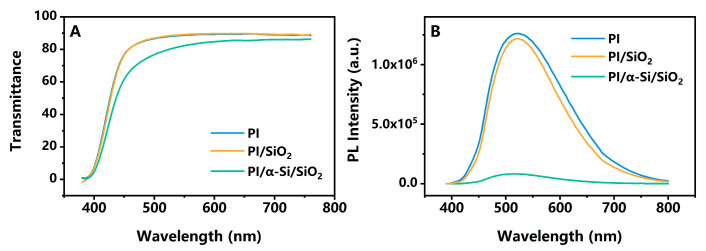
(**A**) UV-vis and (**B**) PL spectrum of three samples: blue lines, PI; orange lines, PI/SiO_2_; green lines, PI/α-Si/SiO_2_; excitation wavelength at 380 nm.

PI/SiO_2_ is the most typical structure in TFT to realize flexibility and serve as a buffer layer to reduce the impact of subsequent processes on the back of the channel. In order to demonstrate the impact of interface polarization, α-Si is also introduced. α-Si can increase the binding force between PI and SiO_2_, thereby enhancing the peel force even in higher temperature and humidity [17]. A stronger binding force means stronger intermolecular forces at the interface, or better interface contact. This has a positive improvement effect on interface polarization [18]. Therefore, a very thin α-Si film, 0.005 μm (5 nm), is adopted between PI and SiO_2_. Meanwhile, it can be easily implemented under the current process and equipment without the need to develop new materials, which means longer time periods and higher material costs. But the transmittance will be satisfied. This is very important for the sensors in phones or computers, especially for full screen display [19]. The transmittance of PI/SiO_2_ is almost the same as pure PI films. The transmittance at 600 nm is 89.34% for PI/SiO_2_ and 89.71% for PI. For PI/α-Si/SiO_2_, it is slightly lower, 84.73%, but is still within an acceptable range (>80%) [20] (Figure 2A). Due to the intramolecular charge transfer in the rigid structure of PI, PI can generate PL [21]. In Figure 2B, it can be seen that there is a marked decrease in intensity for PI/α-Si/SiO_2_, but it is almost the same for PI and PI/SiO_2_. The normalized spectrum has also been attached in Figure S2. This is also influenced by the strong absorption of α-Si [22]. The SEM images of PI/SiO_2_ and PI/α-Si/SiO_2_ are shown in Figure S3. It is difficult to directly observe the presence of a-Si in SEM due to the limitation of equipment. But a more distinct interface can still be identified between PI/SiO_2_, whereas the transition at the interface appears smoother for PI/α-Si/SiO_2_. However, the existence of α-Si can be confirmed via UV-vis and PL.

According to the basic theory of dielectric polarization [23], the polarization can be divided into four categories by response time: electron polarization, ion polarization, dipole polarization, and interface polarization, the speed decreasing from fast to slow. Typically, the slowest interface polarization response speed is between 10^−3^~10^2^ Hz [24]. In other words, it can even last several minutes. This is very consistent with the conditions for image sticking. The existence of interface polarization can be confirmed by impedance spectroscopy (IS) [25].

**Figure 3:**
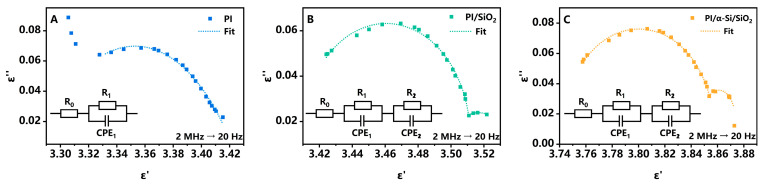
The dielectric spectrum of three samples: (**A**) PI; (**B**) PI/SiO_2_; (**C**) PI/α-Si/SiO_2_. Insets: equivalent circuits diagram for fitting.

The *ε* under alternating electric conditions is complex, composed of *ε′* and *ε″*. Interface polarization, as a typical relaxation polarization, can be described using the Debye theory and its related modifications [26]. In the ideal model,

(1) $$\varepsilon^{\prime }=\varepsilon_{\infty }+(\varepsilon_{s}-\varepsilon_{\infty })\frac{1}{1+{\left(\omega \tau_{1}\right)}^{2}}$$

(2) $$\varepsilon^{\prime \prime }=(\varepsilon_{s}-\varepsilon_{\infty })\frac{\omega \tau }{1+{\left(\omega \tau_{1}\right)}^{2}}$$

where *ε_s_* is the electrostatic dielectric constant at frequencies approaching 0, *ε_∞_* is the dielectric constant at frequencies approaching *∞*, *ω* is the angular frequency of the alternating electric field, and *τ* is the relaxation time. It will appear as a semicircle in the Cole–Cole plots. However, there is a certain deviation between the actual situation and the ideal model. When there are multiple *τ*_1_, and considering the influence of conductivity, it will manifest as multiple “deformed” semicircles [27]. This indicates the existence of multiple polarization mechanisms, as shown in Figure 3. The corresponding Bode plots are presented in Figure S4. In the range of 20 Hz to 2 MHz, PI shows only one kind of polarization that could be observed. The high-frequency tail may be from the current leakage at the edge. The frequency is located at around 1 MHz, indicating that this is a faster polarization process, which can be attributed to dipole polarization in PI. In an equivalent circuit, it can be fitted with an RC parallel circuit and R_0_ resistance, such as wires. However, in PI/SiO_2_ and PI/α-Si/SiO_2_, another polarization occurs at a lower frequency. The polarization of SiO_2_ and α-Si originates from their electron and ion polarization, with a little dipole polarization from defects or impurities, which are usually in the visible or infrared frequency. The response at low frequencies can be reasonably attributed to interface polarization [28]. For PI/SiO_2_, the characteristic frequency is 74 Hz, but for PI/α-Si/SiO_2_, it is 117 Hz. This means that when the electric field changes, the charges at the interface will be slightly delayed and unable to keep up with the changes in the electric field. As a result, hysteresis and image sticking will occur [18]. The data also suggest that the interfacial modification layer can accelerate interfacial polarization, indicating that there will be better image sticking performance. The important influence of interface on hysteresis also exists in perovskite solar cells, and interface modification is also an effective method for the improvement [29]. Indeed, this relatively “slow process” also occurs in other thin-film layer structures within the TFT. For example, studies have shown that deep traps at the channel surface can also lead to significant hysteresis [30]. Therefore, to better mitigate image sticking, various slow-rate processes require careful consideration.

**Figure 4:**
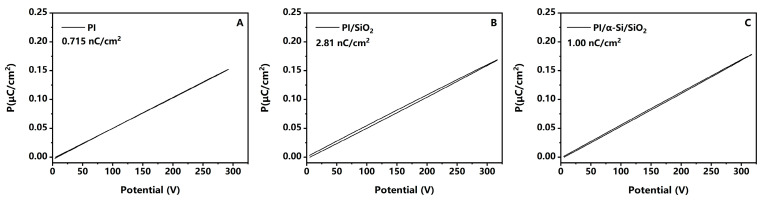
The hysteresis loop of three samples at 0.1 Hz, 50 MV/m. (**A**) PI; (**B**) PI/SiO_2_; and (**C**) PI/α-Si/SiO_2_.

**Figure 5:**
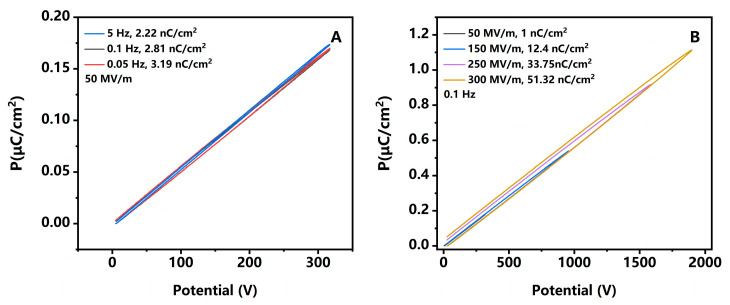
(**A**) The hysteresis loop of PI at 50 MV/m with varying frequency; (**B**) the hysteresis loop of PI/α-Si/SiO_2_ at 0.1 Hz under different field strength.

Ferroelectric analysis is a research method for studying the spontaneous polarization of ferroelectric materials. It can also be applied to studying the polarization of some slower polarization processes under external electric field changes [25,31]. In this article, ferroelectricity is used to confirm the residual polarization of three samples, as shown in Figure 4. In order to effectively distinguish between interface polarization and dipole polarization of PI, further considering the testing time for image sticking, relevant tests are conducted at the frequency of 5 Hz to 0.05 Hz. PI shows little residual polarization, 0.715 nC/cm^2^, at the condition of 0.1 Hz, 50 MV/m. Compared to it, PI/SiO_2_ is larger, 2.81 nC/cm^2^. It can be converted to charge density via basic charge, 1.75 × 10^10^/cm^2^, which is consistent with literature reports [9,32]. This indicates that ferroelectricity is also an effective method for studying image sticking. When the frequencies become lower, the more residual polarization will be generated (Figure 5A). In contrast, the residual polarization of PI/α-Si/SiO_2_ further decreases to 10.0 nC/cm^2^ in the same condition, which is consistent with the faster response rate observed in the dielectric spectrum. According to Formula (5), the voltage or electric field strength will also affect the charges at the interface. However, only PI/α-Si/SiO_2_ can withstand higher voltage without easily experiencing breakdown. Therefore, only relevant data of PI/α-Si/SiO_2_ is displayed in Figure 5B. It gradually increased from 1 nC/cm^2^ to 51.32 nC/cm^2^. This is highly consistent with the fact that the more severe the image sticking under higher brightness, the larger the voltage applied on TFTs.

Typically, the dielectric constant and conductivity of polymers and inorganic material are quite different. When polymers and inorganic material form composite films, the interface between them will generate space charge, which can affect the output currents of TFT. The quantity of space charge can be analyzed through a simple RC (resistance and capacitance) model (similar to the circuits in Figure 3 without the R_0_). In two-layer structures, such as PI/SiO_2_, when the voltage changes, the electric field in the layers will gradually change over time. At this procedure, both conduction current and displacement current exist simultaneously.

(3) $$j=\gamma_{1}E_{1}(t)+\varepsilon_{0}\varepsilon_{s1}\frac{dE_{1}}{dt}=\gamma_{2}E_{2}(t)+\varepsilon_{0}\varepsilon_{s2}\frac{dE_{2}}{dt}$$

(4) $$u=E_{1}d_{1}+E_{2}d_{2}$$

*j* is the total current density, *ε*_0_ is the dielectric permittivity of vacuum, and *γ, ε_s_, E*, and *d* are the conductivity, dielectric constant (specifically, it refers to the static dielectric constant), electric strength, and thickness of PI and SiO_2_. Finally, the charges at the interface, *σ(t)*, can be computed as follows:

(5) $$\sigma (t)=\varepsilon_{0}\frac{\gamma_{1}\varepsilon_{s2}-\gamma_{2}\varepsilon_{s1}}{d_{1}\varepsilon_{s2}+d_{2}\varepsilon_{s1}}u(1-e^{-t/\tau })$$

(6) $$\tau_{2}=\varepsilon_{0}\frac{d_{1}\varepsilon_{s2}+d_{2}\varepsilon_{s1}}{d_{1}\gamma_{2}+d_{2}\gamma_{1}}$$

*τ_2_* is the typical response time of this polarization. According to the above deduction, the combination of material dielectric constant, conductivity, and film thickness will affect the accumulation amount and response time.

**Figure 6:**
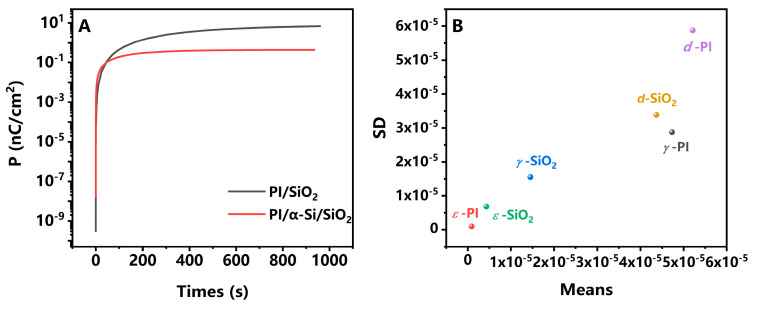
The simulation results of (**A**) the polarization of PI/SiO_2_ and PI/α-Si/SiO_2_; (**B**) MOAT analysis of PI/SiO_2_.

We use simulation methods to further elucidate the role of interface polarization, as shown in Figure 6. The relevant parameters are listed in Table S1. It can be seen that PI/SiO_2_ generates more polarized charges and has a slower response time. The introduction of α-Si not only reduces the generation of charges but also accelerates the response speed of interface polarization. Although the model used has been relatively simplified, considering only the results under ideal conditions, the results are still in good agreement with the dielectric and ferroelectric results. Formula (5) and (6) reveal that there are many factors that affect interface polarization. To further investigate the degree of influence of different factors, the Morris one-at-a-time (MOAT) method was chosen (Figure 6B). MOAT is a relatively efficient method in global sensitivity analysis. Within an acceptable range of accuracy, it can effectively reduce computational complexity [33]. The horizontal axis is the average MOAT value of each input parameter calculated based on the target quantity. A higher average value indicates that the parameter has a significant impact on the aim. The vertical axis represents the standard deviation (SD) of MOAT. It indicates that the corresponding factor has strong interactions with other parameters or exhibits nonlinear effects. Among the three factors of *γ, ε,* and *d* of PI and SiO_2_*,* the *d*_PI and *γ*_PI have the most significant impact on the results. However, the mean is relatively small, so it means that interface modification is a more effective method.

**Figure 7:**
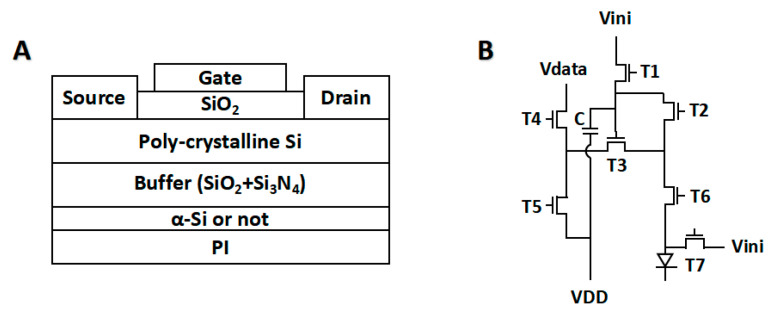
(**A**) The scheme of TFT structure and (**B**) 7T1C pixel circuit are used in tests.

Furthermore, the above theoretical assumptions have been further validated in actual products. The basic structure of TFT and the pixel circuit, 7T1C, used are shown in the figure (Figure 7A,B) [34]. All adopted a relatively universal structure to demonstrate the applicability of the method. In pixel circuits, transistor 3 (T3) is used to control the brightness, while other transistors and capacitors are used to control the writing and erasing of signals. Therefore, T3 is very important for display quality. The quantification of image sticking can be achieved through methods in Figure 1B. T3 is under voltage bias during tests, resulting in interface polarization. The structure of PI/SiO_2_ scores 3.46, but for PI/α-Si/SiO_2_ it is 1.67. The theoretical hypothesis of interfacial polarization demonstrates excellent agreement with experimental observations, confirming the validity of the proposed mechanism. Meanwhile, it also proves that interface modification is an effective improvement measure.

## 4. Conclusions

This study combines experimental and theoretical analysis to elucidate the interfacial polarization mechanism behind image sticking in PI-based flexible OLEDs. The findings reveal that polarization at the PI/inorganic silicon film interface leads to charge accumulation. By incorporating an α-Si interlayer, interface compatibility is enhanced, accelerating polarization response and reducing the residual charge. The close agreement between experimental results and theoretical predictions confirms the validity of the proposed mechanism. More importantly, this work further clarifies the critical role of slow processes in image sticking. We not only advance the understanding of image sticking but also offer a practical approach to optimizing OLED performance, contributing to the development of next-generation flexible display technologies.

## Data Availability

The original contributions presented in the study are included in the article/Supplementary Material, further inquiries can be directed to the corresponding author.

## Supplementary Materials

The following supporting information can be downloaded at: https://www.mdpi.com/article/10.3390/polym17172333/s1, Figure S1: the chemical structure of PI; Figure S2: the normalized PL spectra; Figure S3: the SEM images of PI/SiO_2_ and PI/α-Si/SiO_2_; Figure S4: the Bode plots of impedance spectroscopy; Table S1: The parameter used in simulation.
