# Supplementary material for: Interfacial Polarization Mechanism in Image Sticking of Polyimide-Based Flexible OLEDs
Source: Polymers (Basel). 2025 Aug 28;17(17):2333. doi: 10.3390/polym17172333 (PMC12430894; doi:10.3390/polym17172333)
Supplement: Supplementary file 1 [file polymers-17-02333-s001.zip › polymers-3744991-supplementary.pdf]

Supporting Information

Interfacial Polarization Mechanism in Image Sticking of Polyimide-Based Flexible OLEDs

Zhipeng Li [1,2,*], Haowen Li [2],Dawei Ma [2], Baojie Zhao[2] and Yanbo Li[1]

[1] Institute of Fundamental and Frontier Sciences, University of Electronic Science and Technology of China, Chengdu 611000, China
yanboli@uestc.edu.cn (Li, Y.B.)
[2] Mianyang BOE Optoelectronics Technology Co., Ltd., Mianyang 621000, China;
lihaowen@boe.com.cn (H.W. Li); madawei@boe.com.cn (D.W. Ma); zhaobaojie@boe.com.cn (B.J. Zhao)
* Correspondence: lizhipeng-b11@boe.com.cn (Z.P. Li);

1. The chemical structure of PI

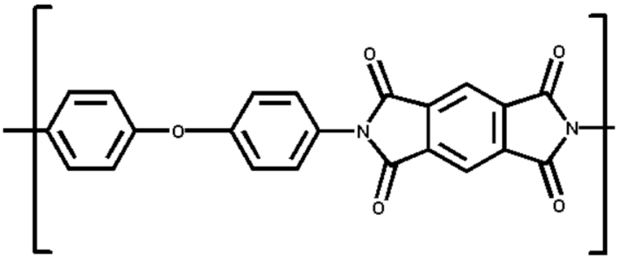

Figure S1: The chemical structure of PI

2. The normalized PL spectra

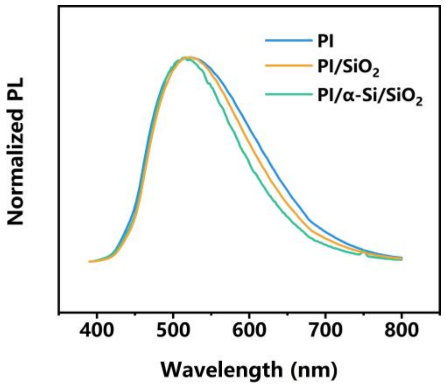

Figure S2: The normalized PL spectra of three samples: blue lines, PI; orange lines, PI/SiO_2; green lines, PI/α-Si/SiO_2.

3. The SEM of PI/SiO_2 and PI/α-Si/SiO_2

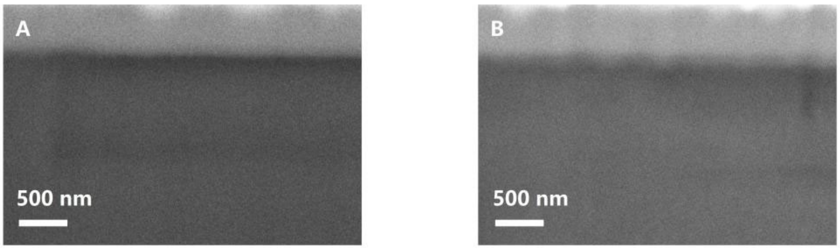

Figure S3: The SEM of (A) PI/SiO_2 and (B) PI/α-Si/SiO_2.

4. The Bode plots of impedance spectroscopy

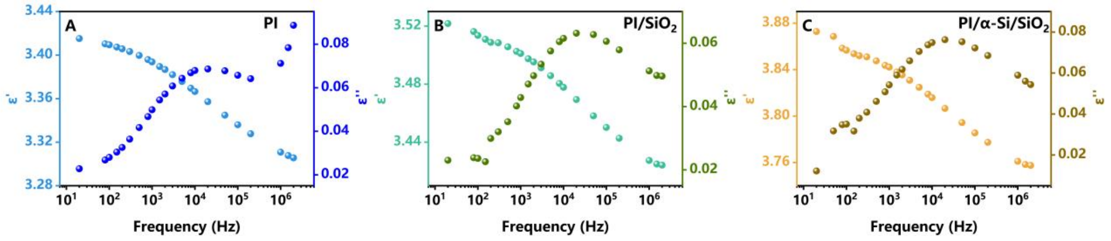

Figure S4: The Bode plots of (A) PI, (B) PI/SiO_2, and (C) PI/α-Si/SiO_2.

5. The parameter used in simulation

layer	*d*/*μm*	*ε*	γ/S·m^{-1}
PI	5.8	3.4	1.5e-12
SiO_2	0.5	4.2	1e-13
α -Si	0.005	12	1e-10

Table S1: The parameter used in simulation
